# A Complicated Case: Presenting Neuropsychiatric Symptoms After COVID Infection

**DOI:** 10.1192/j.eurpsy.2023.1641

**Published:** 2023-07-19

**Authors:** A. Evecen, A. Bülbül, M. T. Karaytuğ

**Affiliations:** 1 Psychiatry, Istanbul Erenkoy Training and Research Hospital for Psychiatry and Neurological Diseases, Istanbul, Türkiye

## Abstract

**Introduction:**

The COVID-19 pandemic has affected millions of people worldwide. Among a large number of deaths, COVID-19 has caused significant health-related sequelae involving many systems of the body. Some results include increased anxiety, depression, insomnia, and other distress symptoms. Additionally, there are reports of sudden onset of psychosis in patients with no psychiatric history following COVID-19.

**Objectives:**

Emphasizing the importance of Covid 19 causing many psychiatric diseases, although its etiology is not yet known.

**Methods:**

Report of a clinical case

**Results:**

We report a 68-year-old previously healthy woman with no personal or family history of mental illness who was hospitalized due to COVID-19 and then started to have psychiatric complaints. She had complaints such as refusal to speak, eat and drink, fear of death which started after her discharge from the hospital due to COVID-19.

On 14.07.2021, she applied to the psychiatry outpatient clinic of our hospital for the first time. In addition to the existing complaints, we have considered the preliminary diagnoses of dementia and psychotic depression due to the increasing fear of being alone, going out alone, confusion in the interim periods, and thoughts that someone will harm her and her family. We consulted the neurology clinic and she was admitted to the neurology service with a preliminary diagnosis of encephalitis. During her hospitalization, the patient’s EEG and MRI were taken, LP was performed, and no pathology was detected in the examinations. Her current psychiatric treatment (escitalopram 10mg/d, medazepam 2mg/d, and olanzapine 2,5mg/d) continued throughout her hospitalization in the neurology service, but her complaints did not change. She was fed formula with an NG tube, IV hydration was provided, and a urinary catheter was inserted.

After ruling out neurological disease on 12.08.2021, the patient was admitted to our psychiatry service with a prediagnosis of catatonic-psychotic depression. Electroconvulsive therapy was planned because the visual hallucinations and persecutory delusions persisted. Total of 7 sessions of ECT were received. The patient’s oral intake started with the current treatment, and her fluent speech began. Olanzapine dose was increased to 7.5mg/d, and escitalopram dose was increased to 20mg/d due to persistent depressive and psychotic symptoms. The patient was discharged on 02.09.2021 with the current treatment. Her complaints had improved before she was discharged.

**Conclusions:**

COVID-19 remains an emerging disease with unknown psychological sequelae. Caution should be exercised regarding psychiatric symptoms and associated risks in patients with a recent diagnosis of COVID-19. Cytokine storm may also be involved in the etiology, in addition to many psychological factors such as fear of illness, uncertainty, and isolation. Potential risk factors and underlying biological mechanisms should be investigated.

**Disclosure of Interest:**

None Declared

